# Supplementary material for: Urine and Serum miRNA Signatures for the Non-Invasive Diagnosis of Adenomyosis: A Machine Learning-Based Pilot Study
Source: Diagnostics (Basel). 2025 Nov 26;15(23):3012. doi: 10.3390/diagnostics15233012 (PMC12691541; doi:10.3390/diagnostics15233012)
Supplement: Supplementary file 1 [file diagnostics-15-03012-s001.zip › Table S2.pdf]

Table S2. Model evaluation: Classification performance metrics for all machine learning models across four analytical scenarios. Accuracy, precision, recall, and F1 score are reported for Logistic Regression, Decision Tree, Random Forest, and Support Vector Machine (SVM) models in serum- and urine-based comparisons of adenomyosis versus negative and positive control groups.

	Model	Accuracy	Precision	Recall	F1 Score
**Serum: Adenomyosis vs. Negative Controls**	Logistic Regression	0.60	0.47	0.60	0.53
	Decision Tree	0.60	0.47	0.60	0.53
	Random Forest	0.60	0.47	0.60	0.53
	SVM	0.60	0.47	0.60	0.53
**Serum: Adenomyosis vs. Positive Controls**	Logistic Regression	0.88	0.88	0.88	0.88
	Decision Tree	0.71	0.74	0.71	0.72
	Random Forest	0.82	0.68	0.82	0.74
	SVM	0.82	0.68	0.82	0.74
**Urine: Adenomyosis vs. Negative Controls**	Logistic Regression	0.90	0.91	0.90	0.89
	Decision Tree	0.80	0.80	0.80	0.80
	Random Forest	0.90	0.91	0.90	0.89
	SVM	0.90	0.91	0.90	0.89
**Urine: Adenomyosis vs. Positive Controls**	Logistic Regression	0.88	0.90	0.88	0.86
	Decision Tree	0.82	0.68	0.82	0.74
	Random Forest	0.88	0.90	0.88	0.86
	SVM	0.88	0.90	0.88	0.86
