# Supplementary figures and images for: Urine and Serum miRNA Signatures for the Non-Invasive Diagnosis of Adenomyosis: A Machine Learning-Based Pilot Study
Source: Diagnostics (Basel). 2025 Nov 26;15(23):3012. doi: 10.3390/diagnostics15233012 (PMC12691541; doi:10.3390/diagnostics15233012)

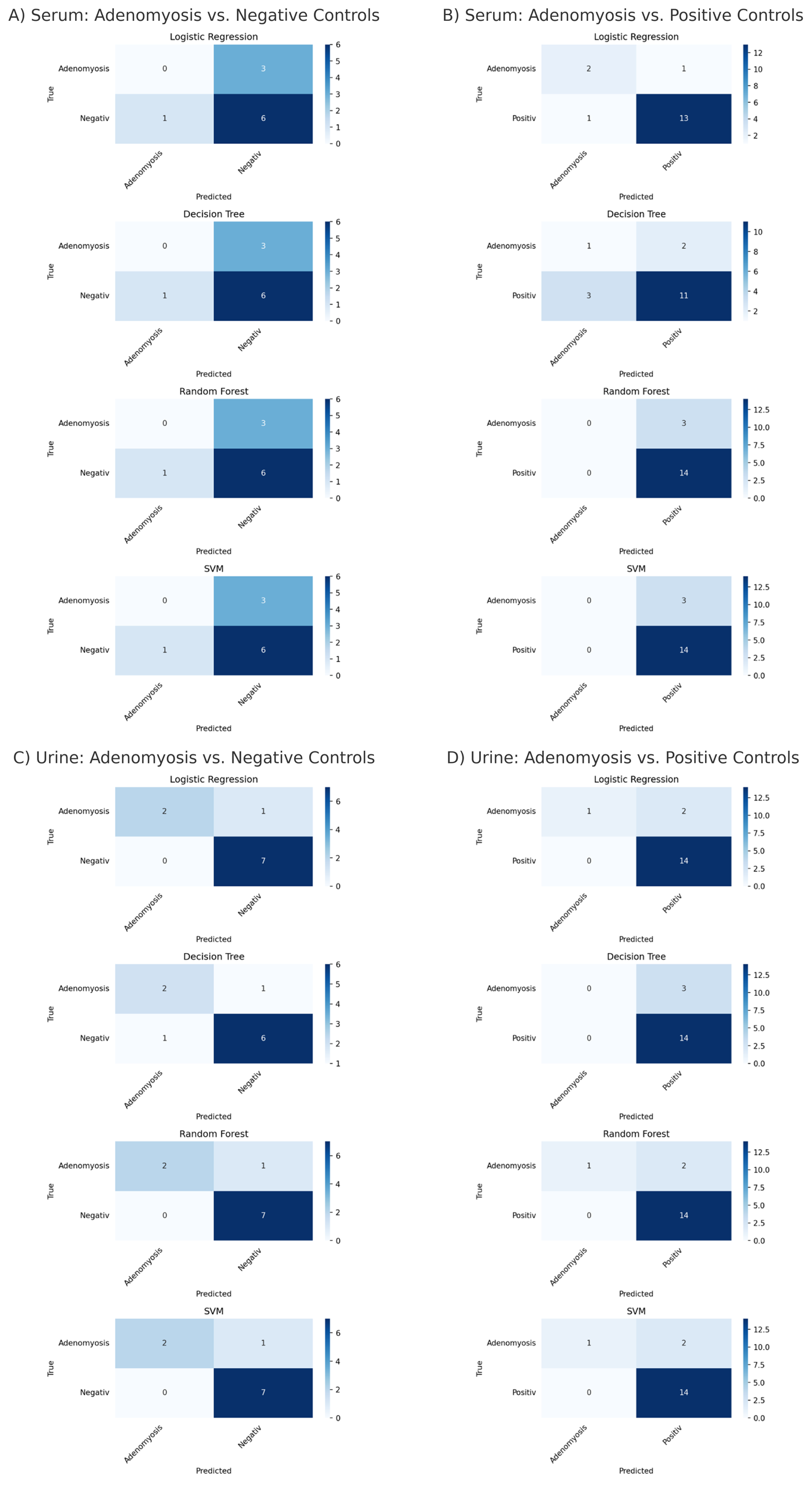

Supplement: Supplementary file 1 [file diagnostics-15-03012-s001.zip › Figure S1. Confusion matrices for all serum- and urine-based classification models.png]
